# Evaluation of two high-throughput proteomic technologies for plasma biomarker discovery in immunotherapy-treated melanoma patients

**DOI:** 10.1186/s40364-017-0112-9

**Published:** 2017-11-10

**Authors:** Su Yin Lim, Jenny H. Lee, Sarah J. Welsh, Seong Beom Ahn, Edmond Breen, Alamgir Khan, Matteo S. Carlino, Alexander M. Menzies, Richard F. Kefford, Richard A. Scolyer, Georgina V. Long, Helen Rizos

**Affiliations:** 1 Faculty of Medicine and Health Sciences, Macquarie University, Sydney, Australia; 2 Melanoma Institute Australia, Sydney, Australia; 3 Australian Proteome Analysis Facility, Macquarie University, Sydney, Australia; 4 Sydney Medical School, University of Sydney, Sydney, Australia; 5 Westmead Hospital, Sydney, Australia; 6 Royal North Shore and Mater Hospitals, Sydney, Australia; 7 Department of Biomedical Sciences, Faculty of Medicine and Health Sciences, 2 Technology Place, Macquarie University, Sydney, NSW 2109 Australia

**Keywords:** Cytokines, Multiplexing, Luminex, Aptamers, SOMAscan, Biomarkers, Liquid biopsies, Immune checkpoint inhibitors, Melanoma

## Abstract

**Background:**

Selective kinase and immune checkpoint inhibitors, and their combinations, have significantly improved the survival of patients with advanced metastatic melanoma. Not all patients will respond to treatment however, and some patients will present with significant toxicities. Hence, the identification of biomarkers is critical for the selection and management of patients receiving treatment. Biomarker discovery often involves proteomic techniques that simultaneously profile multiple proteins but few studies have compared these platforms.

**Methods:**

In this study, we used the multiplex bead-based Eve Technologies Discovery assay and the aptamer-based SomaLogic SOMAscan assay to identify circulating proteins predictive of response to immunotherapy in melanoma patients treated with combination immune checkpoint inhibitors. Expression of four plasma proteins were further validated using the bead-based Millipore Milliplex assay.

**Results:**

Both the Discovery and the SOMAscan assays detected circulating plasma proteins in immunotherapy-treated melanoma patients. However, these widely used assays showed limited correlation in relative protein quantification, due to differences in specificity and the dynamic range of protein detection. Protein data derived from the Discovery and Milliplex bead-based assays were highly correlated.

**Conclusions:**

Our study highlights significant limitations imposed by inconsistent sensitivity and specificity due to differences in the detection antibodies or aptamers of these widespread biomarker discovery approaches. Our findings emphasize the need to improve these technologies for the accurate identification of biomarkers.

**Electronic supplementary material:**

The online version of this article (10.1186/s40364-017-0112-9) contains supplementary material, which is available to authorized users.

## Background

The identification and validation of biomarkers for monitoring disease progression, and predicting response to therapy and patient outcome is a rapidly growing field in cancer research. Cancer patients are currently being treated with new generations and combinations of targeted drugs [1, 2] and immunotherapy [3, 4] but, the activity of these drugs is hampered by variable response rates and the development of treatment resistance [5–7]. For example, approximately 60% of patients with advanced melanoma respond to the combination of the cytotoxic T lymphocyte antigen-4 (CTLA-4) inhibitor, ipilimumab, with an inhibitor of the programmed death-1 (PD-1) receptor (pembrolizumab or nivolumab). However, the benefit of this drug combination comes with significant toxicity and 40% of patients will show no response to therapy [8, 9]. As a result, the identification and validation of reliable, sensitive and accurate predictive biomarkers is necessary for the improved selection and management of cancer patients.

Circulating biomarkers, identified in liquid biopsies such as serum and plasma, can provide an accurate and minimally invasive means for monitoring disease progression, tumor heterogeneity and treatment response. Analysis of certain circulating biomarkers has already yielded important prognostic and diagnostic information, such as prostate-specific antigen (PSA) in prostate cancer [10] and carcinoembryonic antigen (CEA) in colorectal cancer [11], while others have assisted assessment of treatment response and resistance, including circulating tumor DNA (ctDNA) [12, 13] and cancer antigen CA125 [14].

Biomarker discovery using proteomic analysis of liquid biopsies usually involves bead-based multiplex Luminex assays, aptamer-based assays or unbiased mass spectrometry. A literature search using the terms “cancer biomarker” and “liquid biopsy” (August 1, 2017) for publications reporting on liquid biomarker discovery in cancer found 62 publications applying the bead-based system, 36 publications using aptamer-based assays and 252 publications based on mass spectrometry. Although significant attention has focused on cancer biomarker discovery, few studies have compared commonly used protein detection and quantitation platforms.

In this study, we compared a bead-based multiplex assay (Eve Technologies 65-plex Human Cytokine/Chemokine Discovery assay) and an aptamer-based technology (SomaLogic SOMAscan assay) for biomarker discovery in 47 plasma samples derived from 24 melanoma patients treated with combination pembrolizumab and ipilimumab therapy. We show that these widely used assays have limited correlation in relative protein quantification, and this was largely due to differences in specificity and the dynamic range of protein detection. Further, we found that discrepancies in protein quantification and detection were more apparent when two different detection reagents (i.e. antibodies vs. aptamers) were used. Our findings highlight significant limitations in two common approaches for biomarker discovery, and underscore the need for robust method validation and independent assay assessment for blood-based biomarker discovery.

## Methods

### Patients, treatment and clinical assessment

This study included 24 metastatic melanoma patients treated with immunotherapy at Westmead Hospital and Melanoma Institute Australia between July 2014 and December 2015. Patients were treated with pembrolizumab in combination with ipilimumab, administered according to the schedule in the MK3475–029 clinical trial (NCT02089685). Informed consent was obtained from all patients under approved human research ethics committee protocols from the Royal Prince Alfred Hospital.

**Fig. 1:**
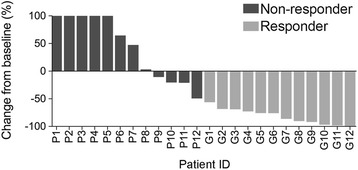
RECIST response of melanoma patients. Melanoma patients were divided into responding (*n* = 12) or non-responding (*n* = 12) groups based on RECIST 1.1 (response evaluation criteria in solid tumor) guidelines following the Week 12 computed tomography scan. Patients in the responding group showed RECIST CR (complete response) or PR (partial response) while those in the non-responding group had RECIST SD (stable disease) or PD (progressive disease). The percentage of change in target lesions from baseline to Week 12 is shown

Investigator-determined objective response was assessed radiologically with computed tomography scans 12 weeks after start of treatment. Patients were divided into responders (Response Evaluation Criteria In Solid Tumor; RECIST CR and PR) and non-responders (RECIST SD and PD) based on RECIST 1.1 guidelines [15] (Fig. 1).

Patient characteristics, clinicopathologic and demographic information including mutation status, lactate dehydrogenase (LDH) levels, disease distribution and American Joint Committee on Cancer (AJCC) M stage (7th edition) were collected (Table 1).

**Table 1:** Patient characteristics and treatment outcomes

Responders				
Patient ID	RECIST at Week 12	Baseline LDH (U/L)	PFS (months)^a^	OS (months)^a^
G1	PR	167	Response ongoing	Alive
G2	PR	215	Response ongoing	Alive
G3	PR	159	Response ongoing	Alive
G4	PR	217	Response ongoing	Alive
G5	PR	185	Response ongoing	Alive
G6	PR	206	Response ongoing	Alive
G7	PR	268	Response ongoing	Alive
G8	PR	167	Response ongoing	Alive
G9	PR	227	Response ongoing	Alive
G10	PR	187	Response ongoing	Alive
G11	PR	140	Response ongoing	Alive
G12	CR	150	Response ongoing	Alive
Non-responders				
Patient ID	RECIST at Week 12	Baseline LDH (U/L)	PFS (months)^a^	OS (months)^a^
P1	PD	303	2.7	5.8
P2	PD	568	2.7	5.1
P3	PD	329	2.7	3.1
P4	PD	300	1.1	Alive
P5	PD	271	0.5	Alive
P6	PD	267	2.7	Alive
P7	PD	169	2.8	Alive
P8	SD	226	4	Alive
P9	SD	269	4.1	Alive
P10	SD	176	2.7	21.7
P11	SD	195	Response ongoing	Alive
P12	SD	402	5.5	8.3

Abbreviations: *PR* partial response, *SD* stable disease, *PD* progressive disease, *CR* complete response, *LDH* lactate dehydrogenase, *PFS* progression free survival, *OS* overall survival, *U/L* units per liter

^a^Data cut off on 30th April, 2017. 11 out of 24 patients had progressed at time of analysis, all of whom were in the non-responders group. Median follow-up for these patients is 24.3 months. Normal range of LDH is 120–250 U/L

### Plasma samples

Peripheral blood samples (~10 ml) were collected from patients in EDTA vacutainer tubes (BD Vacutainer Blood Collection Tubes) before treatment (baseline biopsy) and early during therapy (EDT; collected between 1 to 6 weeks after treatment initiation). All patients had an available EDT sample and 23 patients also had a matched baseline biopsy. Blood samples were centrifuged at 1500 rpm (800 x g) for 15 min at room temperature to separate plasma. Approximately 3–4 ml plasma were collected and centrifuged again at 4100 rpm (1600 x g) for 10 min at room temperature. Clarified plasma samples (1 ml aliquots) were stored at −80 °C, and the plasma volume required for the Discovery (155 μl), SOMAscan (130 μl) and Milliplex (50 μl) assays were obtained from the same plasma aliquot.

### Proteomic assays

Undiluted baseline and EDT plasma samples from 24 patients were profiled using the 65-plex Discovery assay (Human Cytokine Array/Chemokine Array 65-Plex Panel; Cat no: HD65, Eve Technologies, Alberta, Canada) and the 1310 protein SOMAscan assay (SomaLogic, Inc. CO, USA).

The 65-plex Discovery assay is based on the Luminex technology and utilizes the Millipore assay that comprises of fluorescent color-coded beads pre-coated with capture antibodies targeting 65 specific cytokines. Plasma samples were incubated with the beads before the addition of biotinylated detection antibodies followed by phycoerythrin (PE)-conjugated streptavidin. Bound cytokines were identified and quantitated using the Bio-Rad BioPlex 200 bead analyzer consisting of a dual-laser system which i) activates the fluorescent beads to identify the specific cytokine and, ii) excites the PE conjugate to determine the magnitude of fluorescence, which is in direct proportion to reflect the amount of bound cytokine. This assay utilized 150 μl of plasma per run and each run was performed in duplicate; duplicates did not vary by more than 4%.

The SOMAscan assay uses SOMAmer (Slow Off-rate Modified Aptamer) reagents, which consist of modified short DNA sequences that bind specific protein analytes [16]. Plasma samples were incubated with SOMAmer reagents and protein analytes bound to SOMAmer reagents were then biotinylated before capturing the SOMAmer-protein complexes with streptavidin beads. The SOMAmer-protein complexes were detached, and SOMAmer reagents collected and hybridized to complementary sequences on a microarray chip and quantified by fluorescence, which directly correlates with protein amount in the plasma samples. This assay utilized 130 μl of plasma sample per assay run singly. Five patient plasma samples were run in different batches and data showed high concordance (*r* = 0.99, *p* < 0.0001) between batch runs.

Expression of IL-1α, IL-1RA, TNFα and IL-6 in 13 baseline and 15 EDT plasma samples were further validated using the Luminex technology (Milliplex MAP Human Cytokine/Chemokine Panel; Cat no: HCYTOMAG-60 K, Millipore, St. Charles, MO). This bead-based assay is similar to the 65-plex Discovery assay and utilizes fluorescent color-coded beads pre-coated with capture antibodies targeting 4 specific cytokines. Plasma samples were filtered through 0.22 μm spin filters and 25 μl of undiluted plasma was run in duplicates per assay. Duplicates did not vary by more than 5%. Samples were assayed on a robotic liquid handling workstation (epMotion 5075, Eppendorf, Germany) and read with the BioPlex Systems 100 (Bio-Rad) as previously reported [17].

Fluorescence intensity values derived from the Discovery, SOMAscan and Milliplex assays were reported as relative fluorescent units (RFU). Additionally, for the Discovery and Milliplex assays, a protein standard consisting of purified cytokines at known concentrations was included in each batch run; absolute protein concentrations were calculated from the standard curve and reported as pg/ml. Protein standards were not included in the SOMAscan assay and as such, absolute protein concentrations were not determined.

### Statistical analysis

Differential protein expression analysis was performed using the limmaGP module in GenePattern [18]. Comparison between two groups was performed using Mann-Whitney test, correlation analysis using Spearman’s correlation coefficient, and patient characteristics compared using a Chi square test in GraphPad Prism (version 7.02). Principal Component Analysis (PCA) was performed using the ClutVis program [19].

## Results

### Patient response

Twelve patients responded to pembrolizumab in combination with ipilimumab with a complete response (CR, *n* = 1) or a partial response (PR, *n* = 11) and twelve patients had no objective response, i.e. stable (SD, *n* = 5) or progressive disease (PD, *n* = 7). Clinical characteristics across these two response groups were similar for age, sex and disease volume but were significantly different in the known prognostic factors of baseline LDH and AJCC tumor stage (Tables 1 and 2); earlier AJCC stage and normal LDH are associated with good response to combination immunotherapy.

**Table 2:** Summary of patient characteristics

Characteristics	Responders (*n* = 12)	Non-responders (*n* = 12)	*P* value
Age – no. (%)			
> 65	6 (50%)	2 (17%)	0.083
≤ 65	6 (50%)	10 (83%)	
Sex – no. (%)			
Male	9 (75%)	9 (75%)	>0.999
Female	3 (25%)	3 (25%)	
LDH – no. (%)			
> 1× ULN	1 (8%)	8 (67%)	0.0032
≤ 1× ULN	11 (92%)	4 (33%)	
Disease volume – no. (%)			
SPOD > 1000 mm^2^	7 (58%)	10 (83%)	0.178
SPOD ≤ 1000 mm^2^	5 (42%)	2 (17%)	
AJCC tumor stage – no. (%)			
M1a or M1b	6 (50%)	1 (8%)	0.025
M1c	6 (50%)	11 (92%)	
Mutation – no. (%)			
BRAF^V600E/K^	3 (25%)	5 (42%)	0.3865
Non-BRAF^V600E/K^	9 (75%)	7 (58%)	

Abbreviations: *LDH* lactate dehydrogenase, *SPOD* sum of product of diameters, *AJCC* American Joint Committee on Cancer, *ULN* upper limit of normal. The *p* value was calculated using the Chi square test for a two by two contingency table

### Comparison of assay performance

Baseline and EDT plasma samples from the responding and non-responding patients were analyzed for expression of multiple proteins using the 65-plex Discovery assay (Eve Technologies; 65 proteins detected) and the SOMAscan assay (SomaLogic; 1310 detected proteins).

**Fig. 2:**
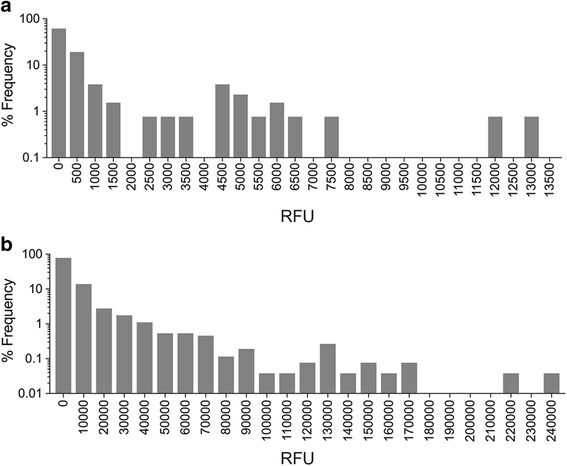
Density distribution of fluorescence intensity values. The distribution, range and frequency of relative fluorescence intensity units (RFU) of proteins detected in all 47 patient samples in the (**a**). Discovery assay and (**b**). SOMAscan assay are shown

The 65-plex Discovery assay has a reported dynamic range of 0.64 pg/ml to 10,000 pg/ml, comparable to other Luminex assays, and a minimal limit of detection (sensitivity) ranging from 0.1 pg/ml to 55.8 pg/ml while the inter-assay variability (coefficient of variation; CV) was between 3.5–18.9% for the 65 cytokines included in the panel [20]. Fluorescence intensity values were detected for every protein in all plasma samples in the Discovery assay, and varied from 46.26 RFU to 13,069 RFU, with a median of 184.7 RFU (Fig. 2a). However, despite a dynamic range across five orders of magnitude, absolute protein concentrations could not be calculated for 15 of the 65 cytokines in more than 75% of plasma samples, as the fluorescence values were below the standard curve (Table 3).

**Table 3:** Detection limits of the Discovery assay

Cytokine	Samples within standard curve^a^	Cytokine	Samples within standard curve^a^	Cytokine	Samples within standard curve^a^
BCA-1	47/47 (100%)	IL-6	43/47 (91%)	**MCP-3**	20/47 (43%)
CTACK	47/47 (100%)	**IL-7**	26/47 (55%)	MCP-4	46/47 (98%)
EGF	38/47 (81%)	IL-8	39/47 (83%)	MDC	47/47 (100%)
ENA-78	47/47 (100%)	IL-9	47/47 (100%)	**MIP-1α**	34/47 (72%)
Eotaxin-1	47/47 (100%)	IL-10	42/47 (89%)	MIP-1β	44/47 (94%)
Eotaxin-2	45/47 (96%)	**IL-12P40**	27/47 (57%)	MIP-1d	47/47 (100%)
Eotaxin-3	47/47 (100%)	IL-12P70	41/47 (87%)	PDGF-AA	47/47 (100%)
FGF-2	47/47 (100%)	**IL-13**	22/47 (47%)	PDGF-BB	46/47 (98%)
**Flt-3 Ligand**	20/47 (43%)	IL-15	46/47 (98%)	RANTES	37/47 (79%)
Fractalkine	42/47 (89%)	IL-16	47/47 (100%)	sCD40L	47/47 (100%)
G-CSF	40/47 (85%)	IL-17A	39/47 (83%)	**SCF**	20/47 (43%)
GM-CSF	40/47 (85%)	IL-18	43/47 (91%)	SDF-1	46/47 (98%)
GRO pan	45/47 (96%)	IL-20	45/47 (96%)	TARC	47/47 (100%)
IFNα2	42/47 (89%)	**IL-21**	22/47 (47%)	TGF-a	36/47 (77%)
IFNγ	36/47 (77%)	IL-23	45/47 (96%)	TNFα	47/47 (100%)
IL-1α	46/47 (98%)	IL-28A	46/47 (98%)	**TNFβ**	22/47 (47%)
**IL-1B**	35/47 (74%)	**IL-33**	29/47 (62%)	TPO	39/47 (83%)
IL-1RA	40/47 (85%)	IP-10	47/47 (100%)	TRAIL	47/47 (100%)
**IL-2**	28/47 (60%)	I-309	47/47 (100%)	TSLP	42/47 (89%)
IL-3	47/47 (100%)	**LIF**	33/47 (70%)	**VEGF-A**	32/47 (68%)
**IL-4**	19/47 (40%)	MCP-1	47/47 (100%)	6CKINE	47/47 (100%)
IL-5	36/47 (77%)	MCP-2	47/47 (100%)		

^a^Percentage of the 47 plasma samples with target protein fluorescence intensity values within the protein standard curve. Absolute cytokine concentration for 15/65 cytokines (shown in bold) could not be calculated in more than 75% of plasma samples because fluorescence intensity values were below the standard curve

Fluorescence intensity values have been shown to be more robust indicators of protein expression compared to absolute concentrations in terms of reproducibility, and for statistical differential analysis [21, 22]. Boxplot graphs showed median fluorescence intensity distributions of the plasma samples were within the range of the standard curve for most of the 65 cytokines in the Discovery assay (Standard 1–7; Additional file 1: Figure S1a and b). However, five of the 65 cytokines (Eotaxin-3, IL-21, IL-3, IL-9 and TSLP) had median fluorescence distributions that were below the standard curve range (Additional file 1: Figure S1b). Detailed analysis of these 5 cytokines showed a symmetrical distribution of RFU (Additional file 1: Figure S2), suggesting that these fluorescence values are unlikely to be background artefacts despite the low readings. As such, fluorescence intensity readings for all 65 cytokines were used in our analyses instead of derived concentrations.

The SOMAscan assay has a larger dynamic range compared to the Discovery assay, detecting protein level from fM to μM across eight orders of magnitude. The average minimal limit of detection is 1.6 pg/ml and the CV varied between 2.9–12.6% for all 1310 protein analytes [23]. Fluorescence data for all 1310 proteins were detected in all plasma samples, and ranged from 45.33 RFU to 238,857 RFU, with a median of 1254 RFU (Fig. 2b). Protein standards of known concentrations were not included for the analytes in the SOMAscan assay, thus absolute protein concentrations could not be calculated and fluorescence data were used instead.

It is important to mention that although fluorescence intensity readings reflect relative protein quantity, they are not directly comparable across different analytes in the SOMAscan assay. For example, a two fold increase in RFU values does not indicate a two fold increase in protein quantity.

### Comparison of protein identification and quantification

**Fig. 3:**
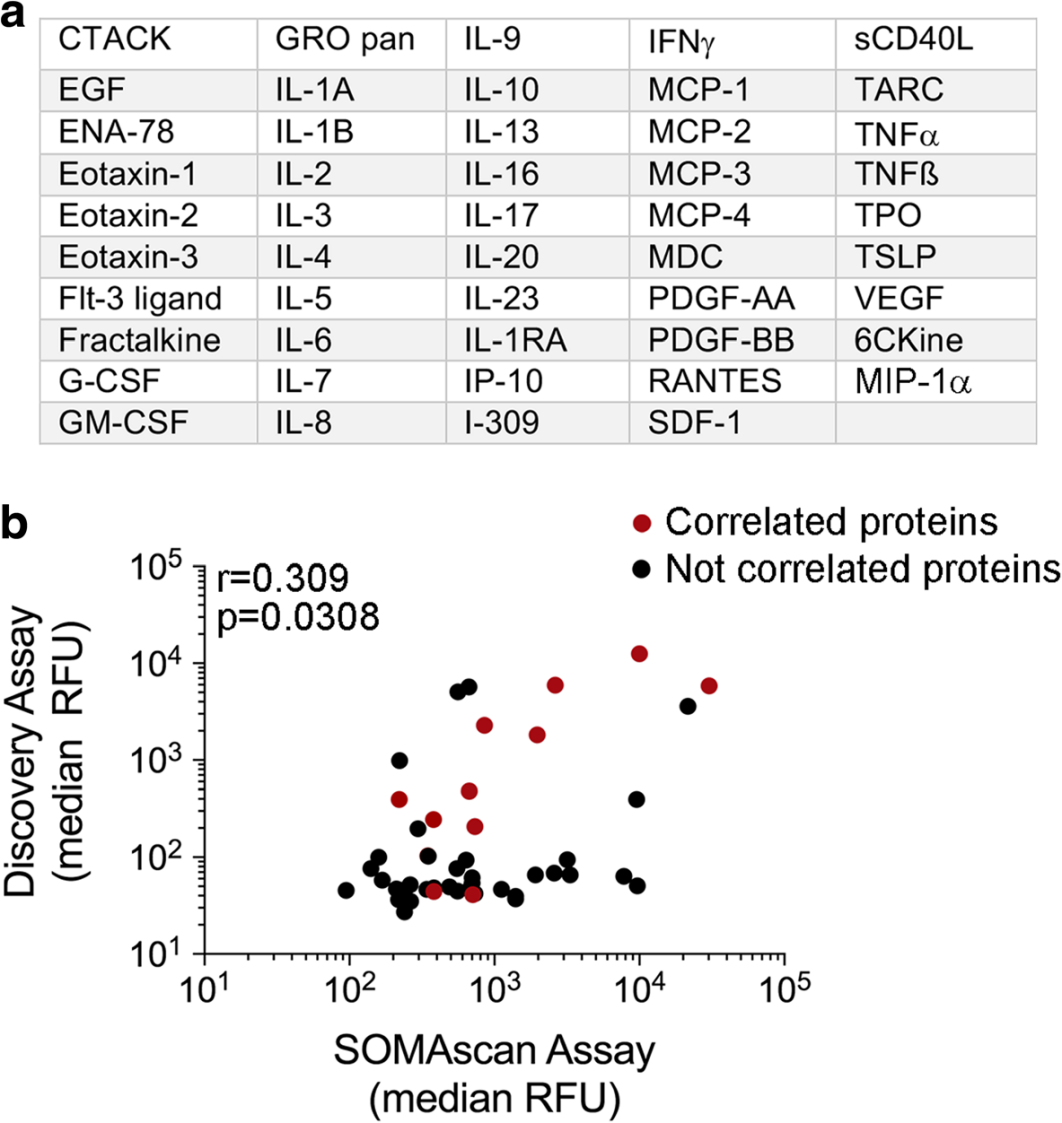
Correlation of common protein targets. **a** List of the 49 proteins shared by the SOMAscan and Discovery Assays. **b** The median relative fluorescence units (RFU) of each of the 49 proteins in all 47 patient samples were derived from the SOMAscan and Discovery assays and tested for correlation (Spearman’s rank correlation coefficient; *r* = 0.3165, *p* = 0.0267). Twelve of the 49 proteins (represented in red) showed significant positive correlation between the two assays when tested individually using Spearman’s rank correlation coefficient (results of the statistical analysis shown in Table 4)

The Discovery and SOMAscan assays have 49 proteins in common (Fig. 3a) and the fluorescence intensity values for each of these 49 proteins were median collapsed and analyzed for correlation.

**Fig. 4:**
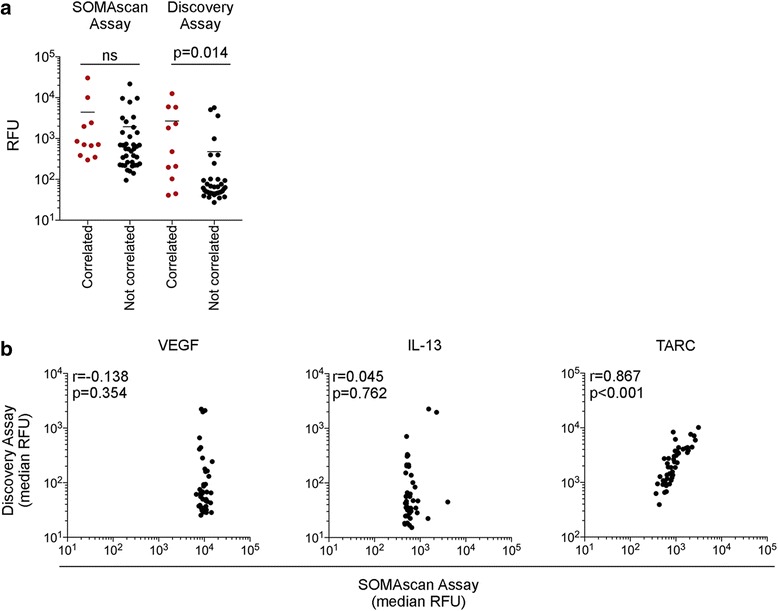
Sensitivity and range of detection of the protein assays. **a** Scatter plot of RFU values for the 49 shared proteins comparing correlated and non-correlated proteins in the Discovery and SOMAscan assays. The RFU values between the correlated and non-correlated proteins in each assay were compared using a Mann-Whitney test (**b**). Median RFU of VEGF, IL-13 and TARC from the SOMAscan and Discovery assays were tested for correlation using Spearman’s rank correlation coefficient; only TARC showed significant positive correlation (*r* = 0.867, *p* < 0.001). Correlation graphs of the non-correlated, high abundance VEGF and low abundance IL-13 showed limited range of RFU in the SOMAscan assay compared to the Discovery assay, in contrast to the correlated TARC protein

As shown in Fig. 3b, the Discovery and SOMAscan median expression data for the 49 shared proteins in the PRE and EDT clinical samples were weakly correlated (*r* = 0.309, *p* = 0.0308, *n* = 47 plasma samples). However, when compared individually, only 12 of the 49 shared proteins showed significant positive correlation between these two assays (both PRE and EDT samples were compared; Table 4). The lack of correlation did not appear to reflect relative fluorescence readings as both high (CTACK and Eotaxin-1) and low level (IL-7 and I-309) proteins were not significantly correlated in these assays (Additional file 1: Figure S3). We noted that nearly all of the 37 proteins that did not correlate between the two assays showed one of two features. The proteins had low RFU in the Discovery assay (*p* < 0.01, Fig. 4a) and/or the proteins showed limited range of RFU in the SOMAscan assay when compared to the equivalent RFU range of the Discovery assay (Fig. 4b). For instance, 30 out of the 37 proteins (81%) that were not correlated had a median RFU of less than 100 in the Discovery assay, and in the SOMAscan assay, 27 out of the 37 proteins (73%) that were not correlated showed limited RFU distribution compared to the Discovery assay.

**Table 4:** Correlation analysis of Discovery and SOMAScan assays

Cytokine	*r* value	*P* value^a^	Cytokine	*r* value	*P* value^a^	Cytokine	*r* value	*P* value^a^
CTACK	0.0242	0.8719	IL-4	0.1467	0.3252	MCP-4	0.1200	0.4218
EGF	0.2815	0.0553	IL-5	0.1427	0.3387	**MDC**	0.5461	<0.0001
ENA-78	0.2833	0.0536	IL-6	0.2487	0.0918	MIP-1α	−0.0028	0.9852
Eotaxin-1	0.2312	0.1179	IL-7	−0.0071	0.9618	**PDGF-AA**	0.7951	<0.0001
Eotaxin-2	0.1752	0.2388	**IL-8**	0.4526	0.0014	**PDGF-BB**	0.6449	<0.0001
**Eotaxin-3**	0.5295	0.0001	IL-9	0.1587	0.2866	RANTES	−0.0445	0.7664
**Flt-3 Ligand**	0.4585	0.0012	IL-10	−0.2023	0.1727	**sCD40L**	0.4882	0.0005
Fractalkine	−0.4095	0.0043	IL-13	0.0454	0.7619	SDF-1	0.2765	0.0599
G-CSF	0.0897	0.5486	IL-16	−0.2042	0.1686	**TARC**	0.8669	<0.0001
GM-CSF	0.1330	0.3727	IL-17A	0.1628	0.2743	**TNFα**	0.3373	0.0204
**GROpan**	0.4460	0.0017	IL-20	−0.3942	0.0061	**TNFβ**	0.3215	0.0275
IFNγ	−0.0659	0.6595	IL-23	−0.1467	0.3252	TPO	0.1433	0.3366
IL-1A	−0.0624	0.6767	**IP-10**	0.5764	<0.0001	TSLP	−0.0460	0.7587
IL-1B	0.1263	0.3975	I-309	−0.1289	0.3877	VEGF	−0.1383	0.3539
IL-1RA	0.0253	0.8661	MCP-1	0.0614	0.6818	6CKine	0.1104	0.4602
IL-2	−0.467	0.0009	MCP-2	−0.2492	0.0912			
IL-3	0.1065	0.4761	MCP-3	−0.2023	0.1728			

^a^Spearman’s rank correlation coefficient test. Only 12 (shown in bold) of the 49 common proteins showed significant positive correlation between the Discovery and SOMAScan assays (*p* < 0.05)

**Fig. 5:**
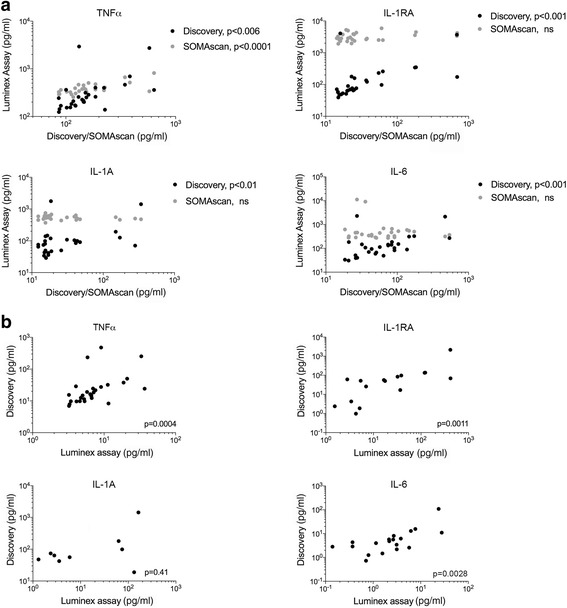
Correlation of four protein targets in bead-based and aptamer-based assays. **a** The relative fluorescence units (RFU) of each of the 4 proteins (IL-1A, IL-1RA, TNFα and IL-6) in 28 plasma samples were derived from the Milliplex, SOMAscan and Discovery assays and tested for correlation (Spearman’s rank correlation coefficient). **b** The absolute protein concentrations (pg/ml) of each of the 4 proteins (IL-1A, IL-1RA, TNFα and IL-6) in 28 plasma samples were derived from the Milliplex and Discovery assays and tested for correlation (Spearman’s rank correlation coefficient)

To validate the protein data, we assessed expression of four proteins (IL-1RA, IL-1A, TNFα and IL-6) that were measured in both the Discovery and SOMAscan assays, and  showed variable expression and correlation data (i.e. only TNFα was highly expressed in the Discovery and SOMAscan assays, whereas IL-1RA, IL-1A and IL-6 were not correlated and showed moderate to low expression). These four circulating proteins were assessed in 28 plasma samples from a subset of the same patients (*n* = 15) using the MAP Human Cytokine/Chemokine Milliplex assay. Fluorescence intensity values for each of the 4 proteins were analyzed for correlation between all three assays. Only TNFα was significantly correlated between all assays while IL-1RA, IL-1A and IL-6 showed significant positive correlation between the Milliplex assay and the Discovery assay but not the SOMAscan assay (Fig. 5a).

Because absolute protein concentrations could be derived for the four proteins from the Discovery and Milliplex assays, we additionally correlated these values and found significant positive correlation for TNFα, IL-1RA and IL-6 (Fig. 5b). Absolute concentration values of IL-1A from the Discovery and Milliplex assays were not significantly correlated, however, it is important to point out that these values could not be calculated from the Milliplex assay in more than 65% of plasma samples as their fluorescence intensities were below the standard curve.

### Identification of differentially expressed proteins in immunotherapy response

**Fig. 6:**
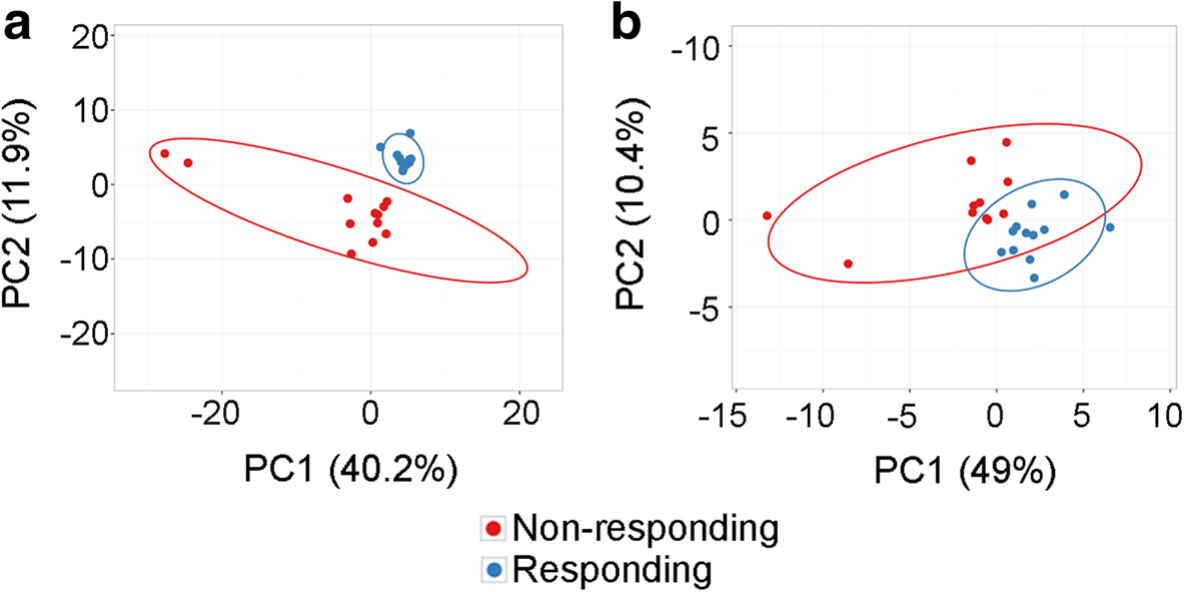
Principal Component Analysis (PCA) of differentially expressed proteins. PCA plots of the (**a**) 178 differentially expressed protein in EDT plasma samples and (**b**) 32 differentially expressed proteins altered in response to immunotherapy showed good separation between the responding (blue) and non-responding (red) patients

We performed differential expression analyses to identify circulating proteins predictive of response to immunotherapy. The PRE and EDT plasma samples were grouped into either response (*n* = 12 for PRE, *n* = 12 for EDT samples) or non-response (*n* = 11 for PRE, *n* = 12 for EDT samples) groups and differential expression between the two groups was analyzed using LimmaGP. From the 1310 proteins in the SOMAscan assay, 178 were differentially expressed in EDT plasma samples (q < 0.25, *p* < 0.05), whereas no proteins were differentially expressed in the baseline samples between the two response groups. 175 out of the 178 differentially expressed proteins were expressed higher in the non-response group and PCA analysis of the differentially expressed proteins indicated clear separation of the EDT plasma samples between the two groups (Fig. 6a). In contrast, comparison of the fluorescence intensity values of the 65 cytokines from the Discovery assay did not reveal any significant differences at baseline or EDT. Of the 178 differentially expressed proteins in EDT samples from the SOMAscan assay, five were part of the Discovery assay panel. However, the five proteins (EGF, SDF-1, CTACK, IL-20 and IL-6) were not positively correlated between the two assays (Table 4).

We also compared changes in proteins in response to therapy and assessed whether these changes predicted response. For this analysis, EDT RFU values were subtracted from the matched baseline RFU data. None of the 65 cytokines from the Discovery assay showed significant differences between the two patient groups in response to therapy. However, we found that 32 SOMAscan proteins were differentially altered on therapy in the response versus non-response groups (q < 0.25, *p* < 0.05). These 32 proteins were all higher (i.e. upregulated on therapy) in the non-response group and PCA analysis of the differentially regulated proteins showed some separation between the two response groups (Fig. 6b). Of the 32 SOMAscan proteins that were significantly altered in response to therapy, MIP-1α and IL-3 were included in the Discovery assay panel, but expression data of these from the two assays did not show significant correlation (Table 4).

## Discussion

Despite advances in proteomic technologies and high-throughput analyte detection systems, successful identification and validation of new biomarkers for cancer diagnosis, and for predicting treatment response has been poor. A significant challenge lies in the limitations of current proteomic techniques. For example, mass spectrometry, although offering more accurate identification of proteins, is limited by interference from high abundance proteins. Other technologies such as multiplex bead-based and aptamer-based assays also have limitations imposed by the specificity and potential cross-reactivity of the capture antibodies or aptamers.

In this study, we compared two proteomic techniques widely used in biomarker discovery. We found that the Discovery and SOMAscan assays showed poor correlation in fluorescence data for 49 shared proteins, and as such, these assays did not detect any proteins in common that identified melanoma patients likely to respond to immunotherapy. The lack of correlation appears to be associated with low detection levels in the Discovery assay and/or restricted range of detection for the SOMAscan platform. To gain further insights into the variability between these assays, we re-quantitated four common cytokines using the Milliplex assay, which also utilizes the bead-based Luminex technology. As expected, all four re-tested cytokines were significantly correlated between the Milliplex and Discovery assays. In contrast, three of four re-tested cytokines (i.e. IL-1RA, IL-1α and IL-6) were not correlated between the Luminex and SomaLogic platforms, and importantly these three cytokines showed a limited range of RFU in the SOMAscan assays. These data strongly suggest that the variability between the Discovery and SOMAscan assays reflect poor specificity and sensitivity of many of the capture antibodies or aptamers.

Our analysis of the data from the Discovery assay did not yield any proteins reflective of response to immunotherapy. However, from the SOMAscan assay, 178 proteins were differentially expressed early during therapy in plasma of patients who responded to treatment compared to those who did not respond, and 32 proteins were upregulated upon treatment in patients who did not respond to immunotherapy. These differentially expressed proteins could represent potential predictive biomarkers of immunotherapy response but given the poor concordance between the Discovery and SOMAscan assays, these targets need to be further validated using separate proteomic approaches and in a larger patient cohort. The complete list and analysis of differentially expressed proteins will be reported as part of another study.

It is also worth noting that there may be more value in using the fluorescence intensity data to identify potential targets during differential expression analysis. To support this, we observed that the mean fluorescence values of the lower standards (S1 and S2, Additional file 1: Figure S1) of some cytokines (i.e. SDF-1 and MIP-1a) did not separate well. This suggests decreased sensitivity in detection of these low-level cytokines, and in turn, may contribute to errors in absolute concentration calculation for each of these cytokines as their standard curve will be skewed. However, this effect will not impact analysis using fluorescence values alone.

## Conclusions

Our study highlights inadequacies in two proteomic platforms commonly used for biomarker discovery, which up till now, have not been evaluated extensively side by side. We show that each technique had specific limitations including sensitivity and specificity of the aptamers and antibodies, which may impede biomarker discovery, and this is particularly relevant as these two assays are routinely applied for this purpose. Our findings underscore the critical need for sensitive, accurate and reproducible protein detection systems, and although the application of multiple, independent detection platforms could be beneficial for discovery, this is not always possible or practical. In particular, the proteomic approaches described in this report are expensive and it is not always possible to apply multiple detection methods when analyzing limited and unique patient biopsies. Further, discordant results between assays, as shown in this study, may necessitate additional measurements of the target proteins.

Method development and validation is essential for the field of biomarker discovery. For instance, the identification of all plasma proteins bound to each SOMAmer reagent by liquid chromatography and tandem mass spectroscopy is an ongoing process that will validate the specificity of the aptamers. Indeed, this has recently resulted in the removal of five aptamer reagents due to non-specific protein enrichment from human plasma and protein preparations. Similarly, although individual detection antibodies used in bead-based assays are tested for cross-reactivity, this is limited to reactivity against a restricted panel of antigens. At present, we rely on a series of modern proteomic methods each with significant limitations that hamper the rapid and accurate identification of novel biomarkers. Investment in improving and advancing these technologies is critical to increasing the effectiveness and value of proteomic biomarker discovery.

## Additional file

Additional file 1:
**Figure S1.** Distribution plots of the 65 cytokines targeted by the Discovery assay. Boxplot graphs showing relative fluorescence intensity (RFU; y axis) of the plasma samples (X) for (**a**), 60 of the 65 cytokines that were within the range of the external standards (from known low (S1) to high (S7) concentrations) while (**b**). 5 of the 65 cytokines were out of the standard curve range. Blank (B) values were also included in the assay. **Figure S2.** Distribution plots of five cytokines detected below the standard curve range in the Discovery assay. Histogram graphs showing distribution of the relative fluorescence units (RFU) of 47 plasma samples for Eotaxin-3, IL-21, IL-3, IL-9 and TSLP. **Figure S3.** Correlation of high and low abundance proteins. Median RFU of highly abundant proteins CTACK and Eotaxin-1, and low abundance proteins IL-7 and I-309 from the SOMAscan and Discovery assays were plotted; each point corresponds to a different patient sample (PRE and EDT plasmas). Proteins that are high and low abundance show poor correlation between the two assays. (DOCX 1640 kb)

## Abbreviations

AJCC: American Joint Committee on Cancer
CEA: Carcinoembryonic antigen
CR: Complete response
ctDNA: circulating tumor DNA
CTLA-4: Cytotoxic T lymphocyte antigen 4
EDT: Early during therapy
IL: Interleukin
PCA: Principle component analysis
PD: Progressive disease
PD-1: Programmed cell death 1
PR: Partial response
PRE: Baseline
PSA: Prostate specific antigen
RECIST: Response evaluation criteria in solid tumors
RFU: Relative fluorescence unit
SD: Stable disease
TNF: Tumor necrosis factor
