# Supplementary material for: Evaluation of two high-throughput proteomic technologies for plasma biomarker discovery in immunotherapy-treated melanoma patients
Source: Biomark Res. 2017 Nov 10;5:32. doi: 10.1186/s40364-017-0112-9 (PMC5681837; doi:10.1186/s40364-017-0112-9)
Supplement: Additional file 1: — Figure S1. Distribution plots of the 65 cytokines targeted by the Discovery assay. Boxplot graphs showing relative fluorescence intensity (RFU; y axis) of the plasma samples (X) for (a), 60 of the 65 cytokines that were within the range of the external standards (from known low (S1) to high (S7) concentrations) while (b). 5 of the 65 cytokines were out of the standard curve range. Blank (B) values were also included in the assay. Figure S2. Distribution plots of five cytokines detected below the standard curve range in the Discovery assay. Histogram graphs showing distribution of the relative fluorescence units (RFU) of 47 plasma samples for Eotaxin-3, IL-21, IL-3, IL-9 and TSLP. Figure S3. Correlation of high and low abundance proteins. Median RFU of highly abundant proteins CTACK and Eotaxin-1, and low abundance proteins IL-7 and I-309 from the SOMAscan and Discovery assays were plotted; each point corresponds to a different patient sample (PRE and EDT plasmas). Proteins that are high and low abundance show poor correlation between the two assays. (DOCX 1640 kb) [file 40364_2017_112_MOESM1_ESM.docx]

**Additional file 1**

**
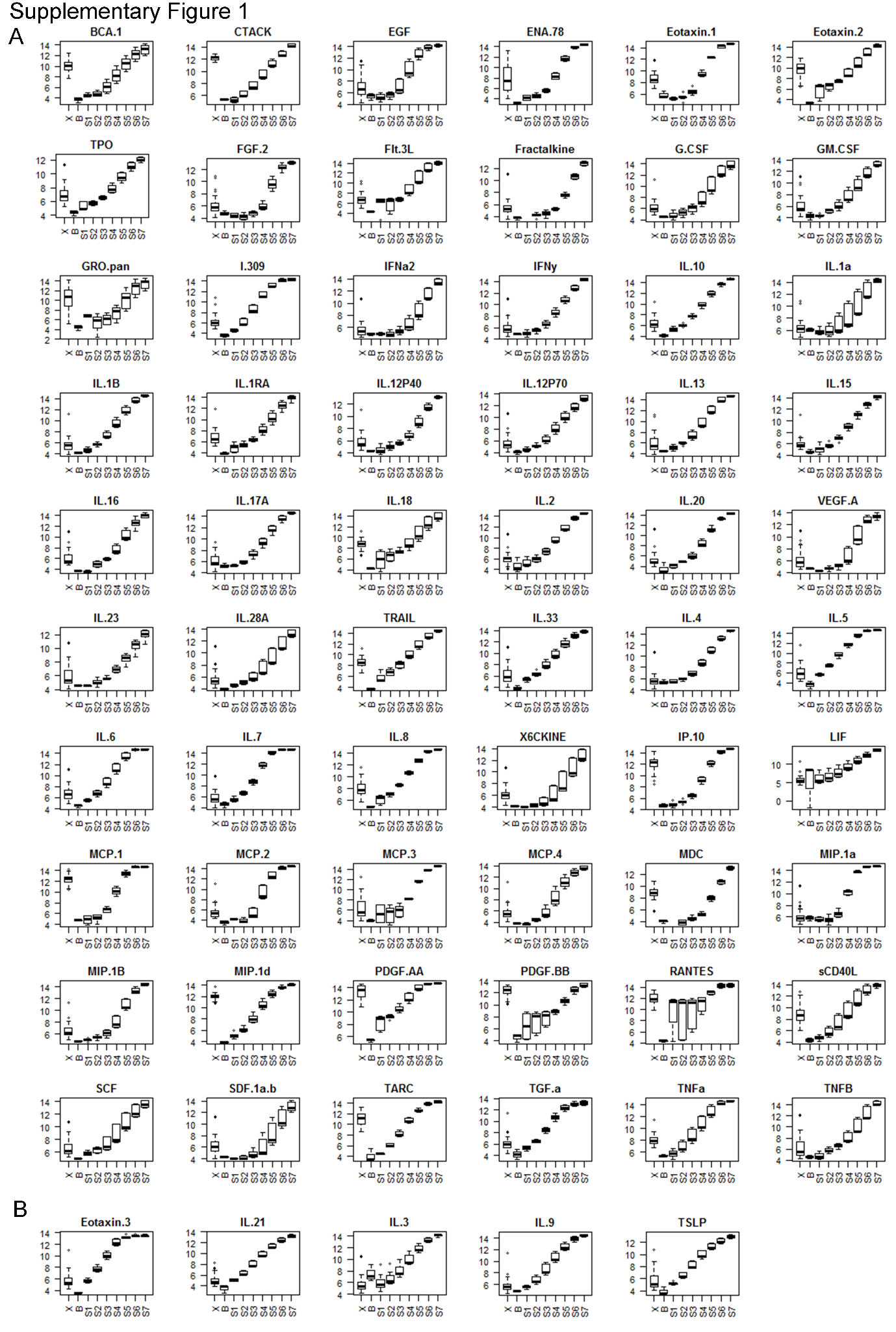
**

**Figure S1: Distribution plots of the 65 cytokines targeted by the Discovery assay.** Boxplot graphs showing relative fluorescence intensity (RFU; y axis) of the plasma samples (X) for **A,** 60 of the 65 cytokines that were within the range of the external standards (from known low (S1) to high (S7) concentrations) while **B.** 5 of the 65 cytokines were out of the standard curve range. Blank (B) values were also included in the assay.

**
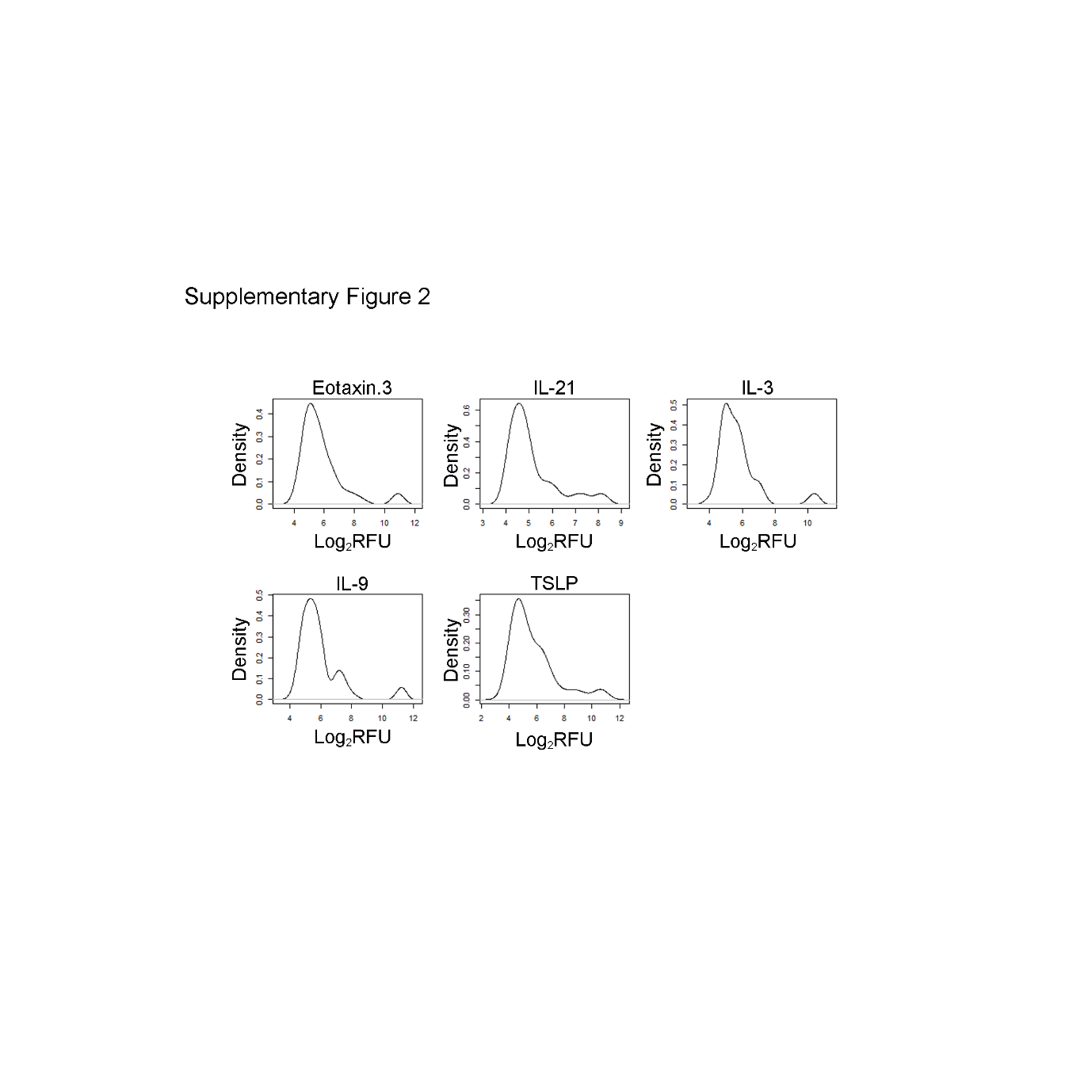
**

**Figure S2: Distribution plots of five cytokines detected below the standard curve range in the Discovery assay.** Histogram graphs showing distribution of the relative fluorescence units (RFU) of 47 plasma samples for Eotaxin-3, IL-21, IL-3, IL-9 and TSLP.

**
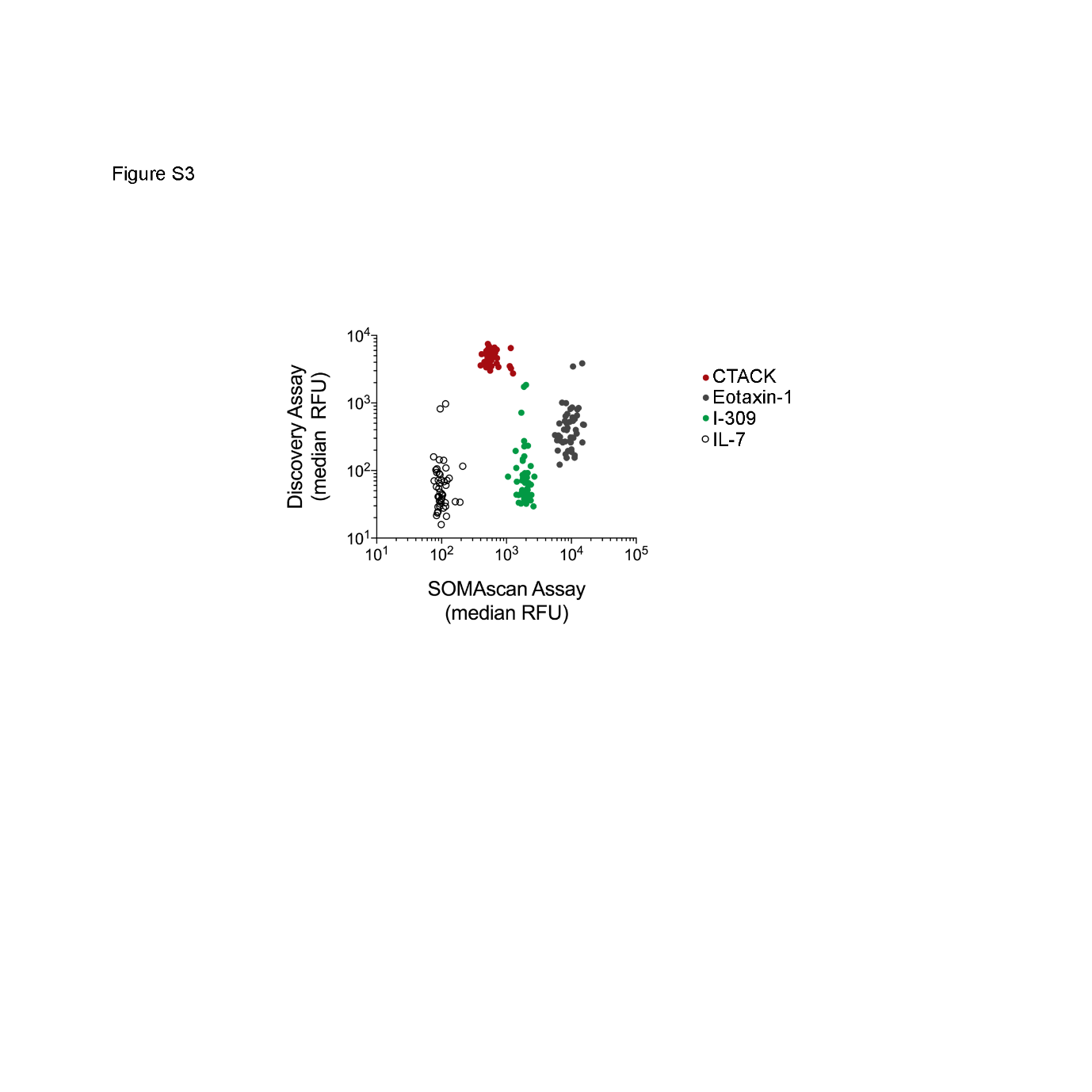
**

**Figure S3: Correlation of high and low abundance proteins.** Median RFU of highly abundant proteins CTACK and Eotaxin-1, and low abundance proteins IL-7 and I-309 from the SOMAscan and Discovery assays were plotted; each point corresponds to a different patient sample (PRE and EDT plasmas). Proteins that are high and low abundance show poor correlation between the two assays.
